# Seizure‐induced impairment in neuronal ketogenesis: Role of zinc‐α2‐glycoprotein in mitochondria

**DOI:** 10.1111/jcmm.15337

**Published:** 2020-04-27

**Authors:** Changhong Tan, Xi Liu, Wuxue Peng, Hui Wang, Wen Zhou, Jin Jiang, Xin Wei, Lijuan Mo, Yangmei Chen, Lifen Chen

**Affiliations:** ^1^ Department of Neurology The Second Affiliated Hospital of Chongqing Medical University Chongqing China

**Keywords:** four β‐subunits of long‐chain L‐3‐hydroxyacyl‐CoA dehydrogenase, ketogenesis, neuron, seizure, zinc‐α2‐glycoprotein

## Abstract

Ketone bodies (KBs) were known to suppress seizure. Untraditionally, neurons were recently reported to utilize fatty acids and produce KBs, but the effect of seizure on neuronal ketogenesis has not been researched. Zinc‐α2‐glycoprotein (ZAG) was reported to suppress seizure via unclear mechanism. Interestingly, ZAG was involved in fatty acid β‐oxidation and thus may exert anti‐epileptic effect by promoting ketogenesis. However, this promotive effect of ZAG on neuronal ketogenesis has not been clarified. In this study, we performed immunoprecipitation and mass spectrometry to identify potential interaction partners with ZAG. The mechanisms of how ZAG translocated into mitochondria were determined by quantitative coimmunoprecipitation after treatment with apoptozole, a heat shock cognate protein 70 (HSC70) inhibitor. ZAG level was modulated by lentivirus in neurons or adeno‐associated virus in rat brains. Seizure models were induced by magnesium (Mg^2+^)‐free artificial cerebrospinal fluid in neurons or intraperitoneal injection of pentylenetetrazole kindling in rats. Ketogenesis was determined by cyclic thio‐NADH method in supernatant of neurons or brain homogenate. The effect of peroxisome proliferator–activated receptor γ (PPARγ) on ZAG expression was examined by Western blot, quantitative real‐time polymerase chain reaction (qRT‐PCR) and chromatin immunoprecipitation qRT‐PCR. We found that seizure induced ketogenesis deficiency via a ZAG‐dependent mechanism. ZAG entered mitochondria through a HSC70‐dependent mechanism, promoted ketogenesis by binding to four β‐subunits of long‐chain L‐3‐hydroxyacyl‐CoA dehydrogenase (HADHB) and alleviated ketogenesis impairment in a neuronal seizure model and pentylenetetrazole‐kindled epileptic rats. Additionally, PPARγ activation up‐regulated ZAG expression by binding to promoter region of *AZGP1* gene and promoted ketogenesis through a ZAG‐dependent mechanism.

## 1 INTRODUCTION

Ketone bodies (KBs) are clearly effective in controlling refractory epilepsy both directly and indirectly.^1^, ^2^ However, whether KB insufficiency exists in epilepsy or seizure, and its mechanism remain unclear. Traditionally, it is believed that neurons could not utilize fatty acids directly, but neurons could utilize KBs generated by liver.^3^ However, neurons were recently reported to utilize fatty acids for β‐oxidation and mainly produce KBs, either under hypoxia or normal conditions.^4^ KBs decrease the vesicular glutamate content and glutamatergic transmission by competing with chloride at the vesicular glutamate transporter.^5^ Acetoacetate (AcAc), one component of KBs, reduces neuronal excitability in the hippocampus by inhibiting voltage‐dependent calcium channels.^6^ Additionally, KBs are associated with altered glutamate metabolism and subsequently increase the synthesis of the inhibitory neurotransmitter γ‐aminobutyric acid (GABA), which inhibits neuronal excitability.^7^ As a glucose‐sparing fuel source, KBs cause hyperpolarization of neurons following glucose deprivation and lead to a reduction in neuronal excitability.^8^ Therefore, it is possible that ketogenesis deficiency in neurons participates in epilepsy or seizure.

Zinc‐α2‐glycoprotein (ZAG) is a lipid‐mobilizing factor that is involved in metabolism,^9^ was recently shown to be localized in both human and rat neurons, and is associated with epilepsy.^10^ Levels of the *AZGP1* (the gene encoding ZAG) mRNA and ZAG protein are decreased in brain tissues from patients with refractory temporal lobe epilepsy and pentylenetetrazole (PTZ)‐kindled epileptic rats compared to controls.^10^, ^11^ Overexpression of ZAG in brain tissues reduces the seizure severity, prolongs the latency of kindling, and alleviates epileptiform discharges of PTZ‐kindled rats,^12^ suggesting an anti‐epileptic effect of ZAG. However, the mechanism has not been clearly elucidated. Interestingly, ZAG plays important roles in lipid metabolism. ZAG overexpression promotes fatty acid β‐oxidation,^13^ while knockdown of ZAG expression significantly promotes lipogenesis and increases the lipid level in hepatocytes.^14^ We speculate that ZAG may exert an anti‐epileptic effect by promoting the β‐oxidation of fatty acids and ketogenesis in neurons. However, whether ZAG promotes fatty acid β‐oxidation and ketogenesis in neurons has not been clearly determined.

It is unknown whether and how ZAG affects ketogenesis. In this study, we used the combined methods of immunoprecipitation and mass spectrometry (IP/MS) and identified an interaction between four β‐subunits of long‐chain L‐3‐hydroxyacyl‐CoA dehydrogenase (HADHB) and ZAG in cultured cortical neurons. HADHB is a mitochondrial protein that facilitates fatty acid β‐oxidation in mitochondria.^15^ However, the effect of ZAG on HADHB activity and ketogenesis in neurons is unknown. Additionally, the mechanism by which ZAG enters mitochondria is also unclear. Our IP/MS assay identified an interaction between ZAG and heat shock cognate protein 70 (HSC70); HSC70 is a molecular chaperone that facilitates the translocation of various proteins into mitochondria^16^, ^17^, ^18^, ^19^ and may facilitate the translocation of ZAG into mitochondria. However, the effect of HSC70 on ZAG translocation into the mitochondria has never been investigated.

Additionally, how neuronal ZAG was decreased in epilepsy or seizure is still unclear. Interestingly, peroxisome proliferator–activated receptor γ (PPARγ) activation increases ZAG levels in the circulation and adipocytes of patients.^20^, ^21^ PPARγ expression is also decreased in the hippocampus of mice with kainic acid–induced seizures.^22^ PPARγ activation suppresses spontaneous recurrent seizures in subjects with pilocarpine‐induced status epilepticus^23^ and exerts a synergistic anti‐epileptic effect with the ketogenic diet on an in vivo seizure model.^24^ Notably, as a transcription factor, PPARγ also promotes fatty acid β‐oxidation and ketogenesis.^25^, ^26^ Therefore, neuronal ZAG decrease in epilepsy or seizure may be mediated by PPARγ, but whether PPARγ regulates fatty acid β‐oxidation and ketogenesis in neurons via ZAG is unclear.

In this study, we focused on the effect of seizure on neuronal ketogenesis and its molecular mechanisms. We found that neuronal seizure model induced by magnesium (Mg^2+^)‐free artificial cerebrospinal fluid (ACSF) and PTZ‐kindled epileptic rats presented impaired ketogenesis, and this impairment could be alleviated by ZAG overexpression. We also identified that ZAG was translocated into mitochondria via a HSC70‐dependent mechanism and then regulated HADHB activity and ketogenesis in neuronal mitochondria. Additionally, we found that PPARγ up‐regulated ZAG expression by binding to the promoter region of the *AZGP1* gene.

## 2 MATERIALS AND METHODS

This study was approved by the Ethics Committee of The Second Affiliated Hospital of Chongqing Medical University (2017‐009).

### 2.1 Animals, AAV transfection and PTZ kindling

Our detailed protocol for animal experiments was reported previously.^12^ Briefly, adult male Sprague‐Dawley rats weighing 200‐300 g (Experimental Animal Center of Chongqing Medical University) were raised in specific pathogen‐free facility with a 12‐hour light/dark cycle and were allowed free access to food and water. An AAV (pHBAAV‐CMV‐ZsGreen) containing the full‐length *AZGP1* cDNA (AAV‐*AZGP1*) or an AAV vector containing only the green fluorescence protein (GFP) cDNA (Vector‐AAV) (Hanbio, Shanghai, China) was stereotaxically injected into bilateral hippocampus (3.0 mm posterior to bregma, 2.0 mm lateral to median line of the skull, and 2.8 mm deep beneath the skull) using a stereotaxia frame (RWD Life Science, Shenzhen, China) after deep anaesthesia with pentobarbital (60 mg/kg, intraperitoneally). After a 3‐week recovery period, rats were killed and brain tissues were sectioned to observe the fluorescence of GFP. Levels of the *AZGP1* mRNA and ZAG protein were also measured to verify the efficacy of AAV transfection and *AZGP1* expression. The rats then received daily intraperitoneal injections of PTZ (35 mg/kg; Sigma‐Aldrich, St. Louis, MO, USA) for 28 days. A modified Racine scale^27^ was used to grade the seizure severity. Rats that presented grade 4 or grade 5 seizures on more than three continuous days were considered successfully kindled and were killed for further experiments. Scalp electroencephalography (EEG) was also performed to evaluate the epileptic discharges in rats received PTZ kindling as we described previously.^12^ PTZ‐induced epilepsy model is mainly for mimicking absence seizure and myoclonic seizure,^28^ and was reported to change the metabolism of fatty acids in cerebral cortex rather than hippocampus,^29^ and KBs were reported not to directly alter the synaptic transmission in seizure models of hippocampal slices and cultured hippocampal neurons.^30^ Therefore, we used cerebral cortex of PTZ‐kindled rats for further experiments.

### 2.2 Primary cortical neuron culture, lentivirus transfection and Mg^2+^‐free ACSF seizure model

Due to the above differences in fatty acid metabolism between cerebral cortex and hippocampus in seizure/epilepsy, primary cortical neuron cultures were prepared from neonatal Sprague‐Dawley rats (both sex) with 24 hours after birth. Briefly, rat brains were removed and the meninges and white matter were stripped. Then, cortices were cut into 1‐mm^3^ particles and digested in DMEM/F‐12 supplemented with 2.5 mmol/L l‐glutamine and 12 mmol/L HEPES (Thermo Fisher, Waltham, MA, USA) containing 2 mg/mL papain (Worthington, Freehold, USA) for 20 minutes at 37°C with gentle shaking every 5 minutes. The brain tissue was gently triturated and filtered, and the filtrate was centrifuged. The precipitated cells were then resuspended in DMEM/F‐12 supplemented with 2.5 mmol/L l‐glutamine and 12 mmol/L HEPES containing 10% horse serum and plated in six‐well plates coated with poly‐l‐lysine (Sigma‐Aldrich) at a density of 2.5 × 10^6^ per well. Cultures were maintained at 37°C in a humidified atmosphere containing 5% CO_2_. All media were replaced with Neurobasal‐A medium (Thermo Fisher) supplemented with 2% B27 (Thermo Fisher) and 1% penicillin‐streptomycin (100 U/mL) (Thermo Fisher) 4 hours later. After 24 hours of culture in vitro, the neurons were incubated with a lentivirus containing the full‐length *AZGP1* cDNA in ubi‐MCS‐3FLAG‐SV40‐EGFP‐IRES‐Puromycin (LV‐*AZGP1*) or LV‐*AZGP1*‐RNAi (GenePharma, Shanghai, China) for 3 days. The LV vectors for LV‐*AZGP1* (Vector‐*AZGP1*) and LV‐*AZGP1*‐RNAi (Vector‐RNAi) containing GFP alone were used as controls, respectively. The effectiveness and efficacy of lentivirus transfection were verified by observing green fluorescence and performing qRT‐PCR and Western blots to determine ZAG levels.

Cultured neurons were incubated with Mg^2+^‐free ACSF containing 145 mmol/L NaCl, 2.5 mmol/L KCl, 10 mmol/L N‐2‐hydroxyethylpiperazine‐N0‐2‐ethanesulfonic acid (HEPES), 2 mmol/L CaCl_2_, 10 mmol/L glucose and 0.002 mmol/L glycine on the 14th day in vitro for 3 hours to establish the neuronal seizure model. Then, the ACSF was replaced with previous culture media and incubated for 24 hours before further experimentation.^31^

### 2.3 Palmitic acid and drug treatments

Cultured neurons were treated with 0, 50, 100, 200 or 500 μmol/L palmitic acid (PA) (P5585; Sigma‐Aldrich) and 1 mmol/L l‐carnitine (LC) (C0158; Sigma‐Aldrich) for 8 hours to establish the appropriate concentration of PA. Neuronal viability was measured as described below. A 100 μmol/L PA treatment was chosen for further experiments assessing KB production. Briefly, LC was dissolved in PBS (1 mol/L). PA stock solutions of 200 mmol/L were prepared in 100% ethanol. Working solutions of 6 mmol/L PA were generated by incubating the PA in PBS containing 10% endotoxin‐free and fatty acid‐free bovine serum albumin at 37°C for 60 minutes. This solution was then added to the media and incubated for 8 hours before KB measurements.

All drugs were dissolved in DMSO. Neurons were incubated with 10 μmol/L pioglitazone (M3283; Abmole, Houston, TX, USA), 10 μmol/L GW9662 (M2748; Abmole) or 5 μmol/L apoptozole (M7575; Abmole) for 24 hours before further experiments. Neurons treated with an equal amount of DMSO served as controls.

### 2.4 qRT‐PCR

Total RNA was extracted with RNAiso plus (Takara, Shiga, Japan) according to the manufacturer's instructions and transcribed into complementary DNAs with HiScript 21II Q RT SuperMix for qPCR (+gDNA wiper) (Vazyme, Nanjing, China) using the Applied Biosystems Veriti‐Well Thermal Cycler (Thermo Fisher) according to the manufacturer's instructions. Quantitative real‐time PCR was performed with the ChamQ Universal SYBR qPCR MasterMix (Vazyme, Nanjing, China) using the CFX96 Real‐Time System (Thermo Fisher). The 2^−ΔΔCt^ method was used to calculate relative gene expression levels.^32^ The primer sequences were provided in Table S3.

### 2.5 Western blot

Total proteins were extracted from cultured neurons or rat brain tissues using RIPA (Beyotime, Haimen, China) containing 1 mmol/L PMSF (Beyotime). Centrifuged protein lysates were mixed and with 5× sodium dodecyl sulfate (SDS) loading buffer and boiled for 10 minutes after determining the concentration using a BCA kit (Dingguo Changsheng Biotechnology, Beijing, China). Equal amounts of protein were separated on 10% SDS‐PAGE gels and transferred to polyvinylidene fluoride (PVDF) membranes (Merck Millipore, Darmstadt, Germany). Membranes were blocked with QuickBlock Blocking Buffer (Beyotime) for 30 minutes at room temperature and then incubated with the following primary antibodies overnight at 4°C: anti‐ZAG antibody (1:300; Santa Cruz, Santa Cruz, CA, USA), anti‐HADHB antibody (1:400; Santa Cruz), anti‐HSC70 antibody (1:3000; Abcam, Cambridge, UK), anti‐COX4 antibody (1:1000; GeneTex, Alton Pkwy Irvine, USA), anti‐GAPDH antibody (1:3000; Proteintech, Wuhan, China) or anti‐β‐actin antibody (1:3000; Proteintech). After washes with Tris‐buffered saline with Tween‐20 (TBST), membranes were incubated with a horseradish peroxidase–conjugated rabbit anti‐mouse antibody (1:3000; Abcam) or mouse anti‐rabbit antibody (1:3000; Abcam) for 1 hour at room temperature. Proteins on the PVDF membranes were visualized using an enhanced chemiluminescence substrate kit (Beyotime) and scanned using a Fusion‐FX7 image analysis system (Vilber Lourmat, Collégien, France).

### 2.6 Chromatin immunoprecipitation

Chromatin immunoprecipitation (ChIP) was performed using a ChIP assay kit (Beyotime) according to the manufacturer's instructions. Briefly, 10^7^ neurons were cross‐linked with 1% formaldehyde at 37°C for 10 minutes, and the reaction was stopped with 0.125 mol/L glycine. Cross‐linked neurons were lysed with SDS lysis buffer, followed by sonication to yield DNA fragments of 100‐500 base pairs. After centrifugation, 200 μL of the supernatant was diluted with 1.8 mL of ChIP dilution buffer containing 1 mmol/L PMSF and cleared with 70 μL of Protein A/G agarose/salmon sperm at 4°C for 30 minutes. The cleared supernatant was then incubated with 2 μg of the anti‐PPARγ antibody (Abcam) or 2 μg of mouse IgG (Beyotime), overnight at 4°C. The immunocomplexes were precipitated with 60 μL of Protein A/G agarose/salmon sperm, eluted, and cross‐links were reversed by incubating the complexes at 65°C for 4 hours. Then, the precipitated DNA was cleaned with RNase and proteinase, and purified with a DNA Purification Kit (Beyotime). The DNA fraction derived from ChIP with the anti‐PPARγ antibody was amplified by qRT‐PCR as described above, using primers of promoter region of *AZGP1* (Table S3). The results of ChIP were analysed using Percent Input Method.^33^

### 2.7 Coimmunoprecipitation and MS

The protein extracts from cultured cortical neurons were diluted in cell lysis buffer for immunoprecipitation (Beyotime) and incubated with 2 μg of mouse IgG (Beyotime), the anti‐ZAG antibody (Santa Cruz), anti‐HADHB antibody (Santa Cruz) or anti‐Hsc70 antibody (Abcam) overnight at 4°C. Then, Protein A/G agarose (Beyotime) was added to the mixtures and rotated at 4°C for 2 hours. The mixtures were centrifuged at 1000 ***g*** for 5 minutes, and the precipitates were washed with lysis buffer five times. Next, the immunoprecipitates were dissolved in 1 × SDS loading buffer and boiled for 10 minutes. Western blots were performed as described above to verify the interactions between proteins. For mass spectrometry, the proteins were separated on SDS‐PAGE gels. After in‐gel digestion, the proteins were analysed using a TripleTOF 5600 (SCIEX, Framingham, USA) for protein identification. MS was performed by BGI Tech (Shenzhen, China). Data of MS are freely available by contacting the authors.

### 2.8 MTS analysis

An MTS analysis was performed using Celltiter 96 AQueous One Solution Assay Kit (Promega, Madison, WI, USA) to identify the appropriate concentration of the PA treatment. Briefly, neurons were seeded into 96‐well plates at density of 6 × 10^4^ cells per well, treated with different concentrations of PA, as described above, and then incubated with 100 μL of new media and 10 μL of MTS in 37°C for 4 hours. The absorption was measured at 490 nm using a microplate reader (MultiSkan, GO; Thermo Fisher).

### 2.9 Immunofluorescence staining

The method for double‐labelled immunofluorescence staining for ZAG in brain tissues and cultured rat cortical neurons was described in detail in our previous studies.^9^, ^10^

For immunofluorescence staining of neuronal mitochondria, cultured neurons in 35 mm glass bottom cell culture dishes (Nest, Wuxi, China) were incubated with MitoTracker Red (25 nmol/L) (Cell Signaling Technology, Danvers, MA, USA) for 30 minutes at 37°C, fixed with cold methanol at −20°C for 15 minutes, permeabilized with 0.1% Triton‐100 for 5 minutes and then washed with PBS three times for 30 minutes to determine the subcellular localization of ZAG in mitochondria. Then, neurons were blocked with 5% normal goat serum (Boster, Wuhan, China) for 1 hour. Thereafter, the cells were incubated with the anti‐ZAG antibody (1:50; Santa Cruz) overnight at 4°C, followed by a FITC‐conjugated goat anti‐mouse IgG (H+L) secondary antibody (1:50; Boster) at room temperature for 1 hour. Finally, the neurons were washed with PBS and stained with DAPI (1:200; Sigma‐Aldrich) for 10 minutes at 37°C.

For cellular localization of ZAG in the brain, fixed brain tissues were dehydrated by 30% sucrose and sliced into 10‐μm‐thick frozen sections. And sections were immersed in acetone for 15 minutes at 4°C, and permeabilized with 0.4% Triton X‐100. Then, the sections were blocked by normal donkey serum (Tianjin TBD Biotechnology, Tianjin, China) at room temperature for 1 hour after antigen retrieval. And sections were incubated with mixed with mouse anti‐ZAG antibody (1:50; Santa Cruz) and rabbit MAP2 antibody (1:100; Abcam) at 4°C overnight. The sections were washed with PBS again and incubated with FITC‐conjugated donkey anti‐mouse (1:50; Proteintech) and Alexa Fluor 555–conjugated donkey anti‐rabbit (1:50; Beyotime) in darkness at room temperature for 1 hour. Then, the sections were stained with DAPI at 37°C for 10 minutes and mounted with antifade mounting medium (P0126; Beyotime).

For cellular localization of ZAG in cortical cultured rat neurons, cultured neurons in 35 mm glass bottom cell culture dishes (Nest) were fixed with 4% paraformaldehyde for 30 minutes at room temperature, incubated with 0.1% Triton X‐100 for 5 minutes, and blocked with 5% normal goat serum (Boster) for 1 hour at room temperature. Then, neurons were incubated with anti‐ZAG antibody (1:50; Santa Cruz) and NeuN (1:200; Merck Millipore) overnight at 4°C. After washed with PBS, neurons were incubated with FITC‐conjugated Goat Anti‐Rabbit IgG (1:200; Proteintech) and Cy3‐conjugated Goat Anti‐Mouse IgG (1:200; Proteintech). Then, DAPI was used to stain nuclei at 37°C for 10 minutes. Images were collected with laser scanning confocal microscopy (Nikon 1R, Japan).

### 2.10 Isolation of mitochondria from neurons

Mitochondria were extracted using the MINUTE Mitochondria Isolation Kit for mammalian cells and tissues with a differential gradient centrifugation strategy according to the manufacturer's instructions.

### 2.11 Measurement of AcAc and BHB levels using the cyclic thio‐NADH method

For KB measurements, cultured neurons were incubated with 1 mL of fresh Neurobasal‐A medium containing 2% B27, 1% penicillin‐streptomycin, 100 μmol/L PA and 1 mmol/L LC per well for 8 hours. Afterwards, the supernatants were collected and measured using the Ketone Body Assay Kit (Sigma‐Aldrich) based on the cyclic thio‐NADH method. The KB levels were normalized to the total cellular protein content (μg) per assay period (hour). 

For measurements of brain tissues, rats were killed and fresh cerebral cortical tissues were dissected, and then, meninges and vessels were stripped. Dissected brain tissues were homogenized by ultrasonication in PBS. After centrifugation, KB levels in the supernatant were measured as described above. KB levels were normalized to the total tissue protein concentration (μg).

### 2.12 Assay of HADHB activity

HADHB activity was analysed using methods described in previous reports.^34^, ^35^ Cortical neurons were sonicated in 100 mmol/L Tris‐HCl (pH 8.3) containing 200 mmol/L NaCl, 0.1% hexamethylphosphoric triamide, 2 mmol/L β‐mercaptoethanol, 0.5 mmol/L EDTA and 0.5% Tween‐20. After centrifugation at 13 000 ***g*** for 10 minutes, the supernatant was used for enzyme assays. The enzymatic activity was measured in 1 mL of 100 mmol/L Tris‐HCl (pH 8.3), 25 mmol/L MgCl2, 100 μmol/L CoA (Sigma‐Aldrich), 40 μmol/L acetoacetyl‐CoA (Sigma‐Aldrich) and 100 μg of cell extract at 30°C for 5 minutes. A microplate reader (MultiSkan, GO; Thermo Fisher) was used to quantify the rate of thiolytic cleavage of acetoacetyl‐CoA at 303 nm. One unit of activity was defined as the amount of enzyme that converted 1 μmol of acetoacetyl‐CoA per minute. Calculations were based on a molar extinction coefficient of 21 400 M^−1^ cm^−1^ at 303 nm.^35^

### 2.13 Statistical analysis

All data are presented as the mean ± standard deviation (SD) from at least three independent experiments. The letter ‘n’ stands for times of repetitive experiments. Data analysis and graph drawing were performed using SPSS 20.0 software (IBM, Armonk, USA) and GraphPad prism 6.01 (GraphPad software, La Jolla, CA, USA). The differences of quantitative data between two groups were analysed using Student's *t* test. The difference of ranked data between two groups was analysed using Mann‐Whitney *U* test. For comparisons of quantitative data between three or more groups, one‐way ANOVA with Bonferroni's or Dunnett's T3 post hoc analysis was used. *P* < 0.05 was considered statistically significant.

## 3 RESULTS

### 3.1 Seizures decreased ketogenesis in neurons, which was reversed by ZAG overexpression

**Figure 1:**
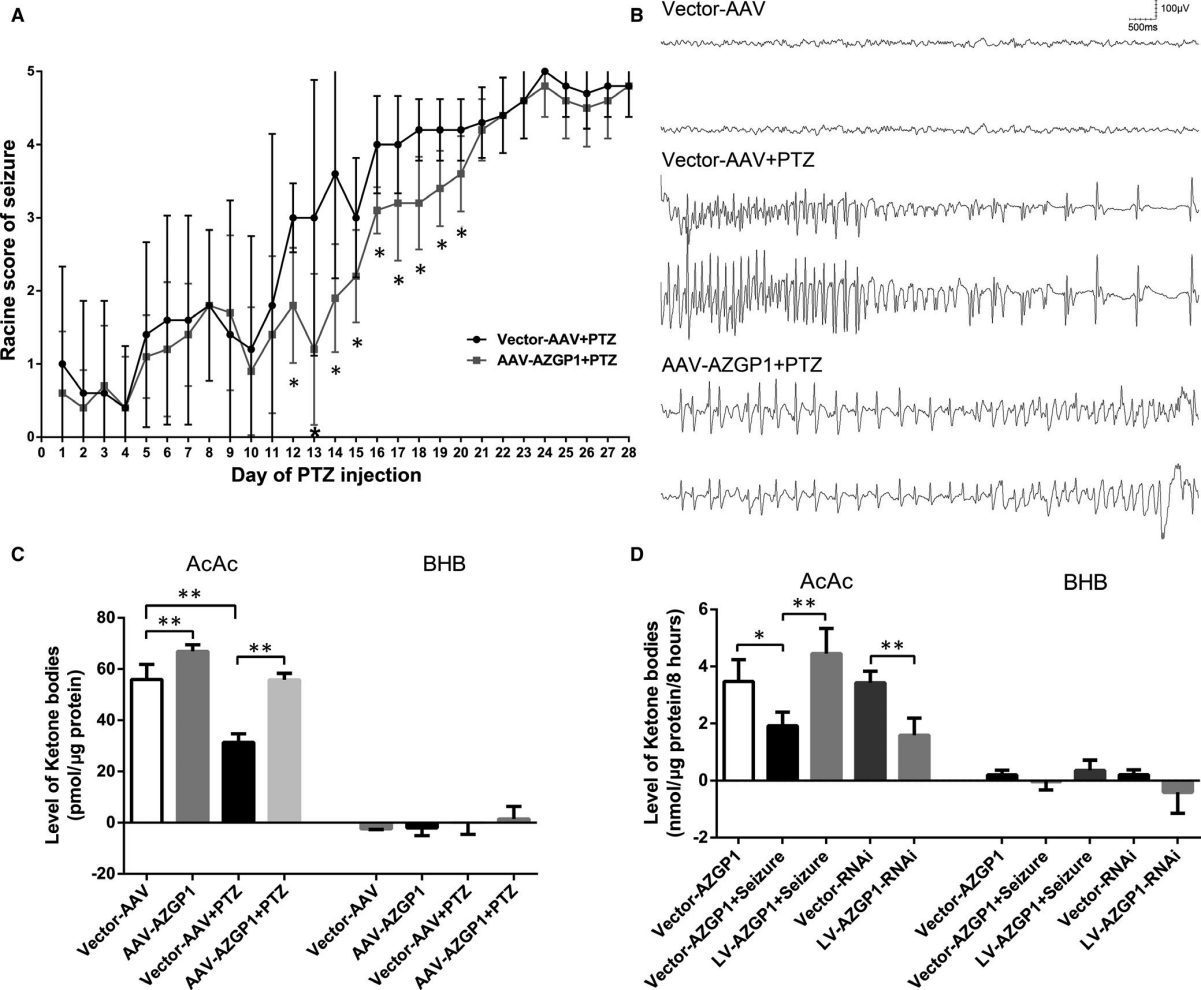
ZAG overexpression alleviated seizure severity and seizure‐induced impairments in ketogenesis. A, After PTZ injection for 12 days, the rats in Vector‐AAV+PTZ group presented significantly severer seizure compared to rats in AAV‐*AZGP1*+PTZ group, and the seizure severity between the 2 groups became similar after PTZ injection for 21 days. B, Scalp EEG showed that AAV‐*AZGP1* treatment decreased the frequency and amplitude of seizure spike wave after PTZ kindling compared to Vector‐AAV treatment. C, KB levels in the cortex of PTZ‐kindled rats and controls. AcAc level was increased in the AAV‐*AZGP1* group compared to Vector‐AAV group. AcAc level was decreased in the Vector‐AAV+PTZ group than in Vector‐AAV group, while increased AcAc level was observed in the AAV‐*AZGP1*+PTZ group than in the Vector‐AAV+PTZ group (n = 4). BHB levels were not different between groups. D, KB levels in the supernatants of cultured primary cortical neurons. AcAc levels in the Vector‐*AZGP1*+seizure group (n = 5) and LV‐*AZGP1*‐RNAi group (n = 5) were significantly decreased compared to the Vector‐*AZGP1* group and Vector‐RNAi group respectively. The seizure‐induced decrease in the AcAc level was rescued by LV‐*AZGP1* treatment (n = 5). BHB levels were not different between groups (* indicates *P* < 0.05, ** indicates *P* < 0.01)

Rats were divided into four groups: Vector‐AAV, Vector‐AAV+PTZ, AAV‐*AZGP1* and AAV‐*AZGP1*+PTZ. The seizure grade of rats in Vector‐AAV+PTZ group and AAV‐*AZGP1*+PTZ group was shown in Figure 1. After PTZ kindling for 12 days, the rats in Vector‐AAV+PTZ group presented significantly severer seizure compared to rats in AAV‐*AZGP1*+PTZ group; however, after PTZ kindling for 21 days, the seizure severity of rats in the two groups became similar (Figure 1A). Scalp EEG showed that AAV‐*AZGP1* treatment decreased the frequency and amplitude of seizure spike wave after PTZ kindling compared to Vector‐AAV (Figure 1B).

AAV‐*AZGP1* group presented increased acetoacetate acid (AcAc) level compared to Vector‐AAV group (66.935 ± 2.588 pmol/μg vs 56.000 ± 5.869 pmol/μg, *P* = 0.010, n = 4). A significantly lower AcAc level was observed in the Vector‐AAV+PTZ group than in Vector‐AAV group (31.334 ± 3.448 pmol/μg vs 56.000 ± 5.869 pmol/μg, *P* < 0.001, n = 4), while a significantly higher AcAc level was detected in the AAV‐*AZGP1*+PTZ group than in the Vector‐AAV+PTZ group (55.866 ± 2.504 pmol/μg protein vs 31.334 ± 3.448 pmol/μg protein, *P* < 0.001, n = 4). However, the β‐hydroxybutyric acid (BHB) level was not different between groups (Figure 1C).

Palmitic acid (PA) and l‐carnitine (LC) must be added to the culture media to measure ketogenesis in neurons.^4^ Considering the potential cytotoxicity of PA towards neurons, a cell viability experiment was performed and 100 μmol/L was chosen as a suitable concentration for the PA treatment. Based on the results of the quantitative real‐time polymerase chain reaction (qRT‐PCR) and Western blot assays, PA and LC treatments did not affect ZAG expression in neurons (Figure S1).

Cultured neurons were divided into five groups: Vector‐*AZGP1*, Vector‐RNAi, Vector‐*AZGP1*+seizure, LV‐*AZGP1*+seizure and LV‐*AZGP1*‐RNAi. After an 8‐hour treatment with PA (100 μmol/L) and LC (1 mmol/L), the AcAc levels were significantly decreased in the Vector‐*AZGP1*+seizure group (1.919 ± 0.483 nmol/μg/8 hours vs 3.474 ± 0.766 nmol/μg/8 hours, *P* = 0.012, n = 5) and LV‐*AZGP1*‐RNAi group (1.592 ± 0.595 nmol/μg/8 hours vs 3.428 ± 0.402 nmol/μg/8 hours, *P* = 0.002, n = 5) compared to the Vector‐*AZGP1* group and Vector‐RNAi group, respectively. The seizure‐induced decrease in the AcAc level was rescued by ZAG overexpression using LV‐*AZGP1* (1.919 ± 0.483 nmol/μg/8 hours vs 4.453 ± 0.887 nmol/μg/8 hours, *P* < 0.001, n = 5). Consistent with results obtained from PTZ‐kindled rats, the BHB level was not different between groups (Figure 1D).

Before measuring KBs in rat brain, AAV‐*AZGP1* was injected into the rat hippocampus to study the effect of ZAG on seizure‐induced impairments in ketogenesis. Both the ZAG protein (0.403 ± 0.109 vs 0.972 ± 0.087, *P* < 0.001, n = 4) and *AZGP1* mRNA (1 vs 2.910 ± 0.296, *P* = 0.008, n = 3) were successfully overexpressed in the cortex compared to rats received the Vector‐AAV injection. Before measuring KBs in cultured neurons, LV‐*AZGP1* or LV‐*AZGP1*‐RNAi was transfected into primary cultured cortical neurons. LV‐*AZGP1* significantly increased the levels of both the ZAG protein (0.506 ± 0.136 vs 1.089 ± 0.080, *P* < 0.001, n = 4) and *AZGP1* mRNA (191.497 ± 34.624 vs 0.941 ± 0.025, *P* = 0.008, n = 3) compared to Vector‐*AZGP1*, while LV‐*AZGP1*‐RNAi decreased the levels of the ZAG protein (0.889 ± 0.102 vs 0.420 ± 0.043, *P* < 0.001, n = 4) and *AZGP1* mRNA (0.899 ± 0.083 vs 0.520 ± 0.068, *P* = 0.004, n = 3) compared to Vector‐RNAi (Figure S2).

### 3.2 Neuronal ZAG expression was decreased by seizures both in vivo and in vitro

Double immunofluorescence staining of rat brain sections revealed the colocalization of ZAG with microtubule‐associated protein 2 (MAP2), suggesting that ZAG was expressed in neurons. This finding was further verified in primary cultures of rat cortical neurons that ZAG colocalized with NeuN. In the cortex of PTZ‐kindled rats, levels of both the ZAG protein (0.235 ± 0.033 vs 0.435 ± 0.031, *P* < 0.001, n = 5) and *AZGP1* mRNA (0.358 ± 0.081 vs 1, *P* = 0.001, n = 4) were decreased compared to the control. Furthermore, in the Mg^2+^‐free ACSF‐induced neuronal seizure model, levels of the ZAG protein (0.577 ± 0.065 vs 0.172 ± 0.073, *P* = 0.002, n = 3) and *AZGP1* mRNA (0.357 ± 0.098 vs 1, *P* < 0.001, n = 3) were also decreased compared to the control (Figure S3).

### 3.3 PPARγ promoted neuronal ketogenesis in cultured neurons via increasing neuronal ZAG expression by binding to the promoter region of the *AZGP1* gene

**Figure 2:**
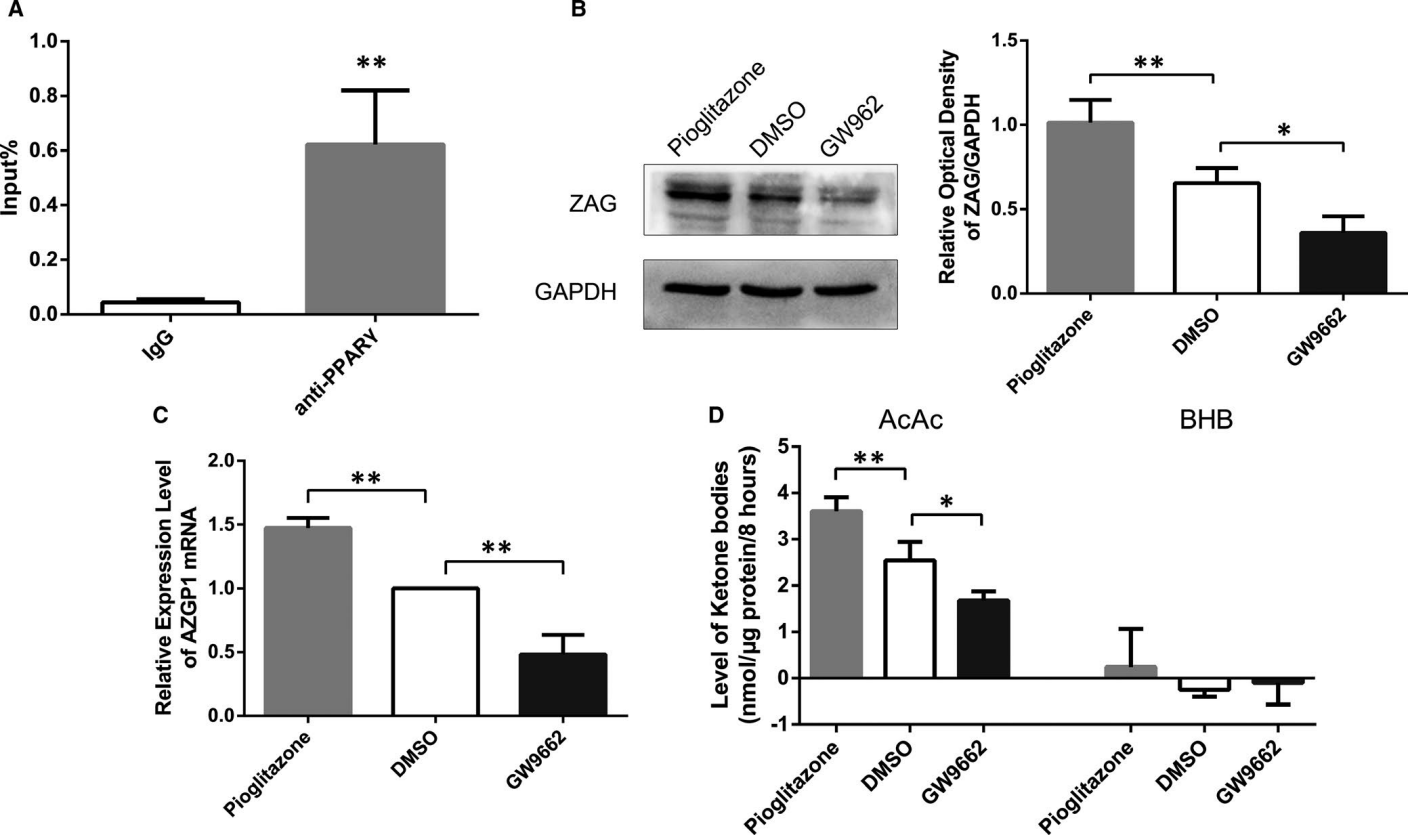
PPARγ promoted neuronal ketogenesis in neurons and increased neuronal ZAG expression by binding to the promoter region of the *AZGP1* gene. A, qRT‐PCR analysis of the immunoprecipitated DNA of anti‐PPARγ and IgG. DNA sequences located in the ZAG promoter region were enriched in the anti‐PPARγ group, but not in the IgG group. Values were expressed relative to per cent input (n = 3). B, Western blots showed that, compared to the DMSO group, pioglitazone increased levels of the ZAG protein (n = 4) and GW9662 decreased levels of the ZAG protein (n = 4). The optical density was normalized to GAPDH levels. C, qRT‐PCR showed that, compared to the DMSO group, treatment with pioglitazone up‐regulated *AZGP1* mRNA (n = 3) levels and GW9662 down‐regulated *AZGP1* mRNA (n = 3) levels. D, AcAc levels in supernatant of cultured primary neurons were increased after treatment with pioglitazone (n = 4) and decreased by GW9662 (n = 4) compared to DMSO. The BHB level was not altered (* indicates *P* < 0.05, ** indicates *P* < 0.01)

Chromatin immunoprecipitation (ChIP) was conducted to examine whether PPARγ regulates ZAG in neurons and its mechanism. The purified PPARγ‐bound DNA sequences obtained from the ChIP experiment were amplified using qRT‐PCR and analysed using Percent Input Method.^33^ The sequence in the ZAG promoter region was enriched in the ChIP group, but not in the IgG group (Input %: 0.044 ± 0.011 for IgG vs 0.622 ± 0.198 for anti‐PPARγ antibody, *P* = 0.007, n = 3) (Figure 2A). The PPARγ agonist pioglitazone and antagonist GW9662 were used to further confirm whether PPARγ regulated ZAG transcription. Cultured primary rat cortical neurons were divided into three groups: pioglitazone (10 μmol/L), GW9662 (10 μmol/L) and dimethyl sulfoxide (DMSO) as a control. Compared to the DMSO group, a 24‐hour treatment with pioglitazone increased the levels of the ZAG protein (1.013 ± 0.135 vs 0.654 ± 0.090, *P* = 0.004, n = 4) and *AZGP1* mRNA (1.474 ± 0.078 vs 1, *P* = 0.003, n = 3) (Figure 2B,C), while GW9662 significantly decreased the levels of the ZAG protein (0.361 ± 0.097 vs 0.654 ± 0.090, *P* = 0.013, n = 4) and *AZGP1* mRNA (0.482 ± 0.153 vs 1, *P* = 0.002, n = 3) (Figure 2B,C). Based on these data, PPARγ regulated ZAG transcription by binding to the promoter region of the *AZGP1* gene. Cultured primary rat cortical neurons were divided into 3 groups to clarify whether PPARγ influenced ketogenesis in cortical neurons: pioglitazone (10 μmol/L), GW9662 (10 μmol/L) and DMSO as a control. After 24 hours of exposure to the agents followed by 8 hours of treatment with PA (100 μmol/L) and LC (1 mmol/L), AcAc level was increased by pioglitazone (3.609 ± 0.298 nmol/μg/8 hours vs 2.543 ± 0.402 nmol/μg/8 6hours, *P* = 0.003, n = 4) and decreased by GW9662 (1.676 ± 0.200 nmol/μg/8 hours vs 0.543 ± 0.402 nmol/μg/8 hours, *P* = 0.010, n = 4) compared to DMSO (Figure 2D). However, the BHB level was not altered (Figure 2D). Thus, PPARγ regulated ketogenesis in neurons.

As PPARγ regulated ZAG expression and ketogenesis, changes in ZAG expression induced by PPARγ activation or inhibition may be a causal factor changes ketogenesis or an independent phenomenon. We investigated the effects of pioglitazone and GW9662 on ketogenesis in neurons after ZAG knockdown or overexpression to clarify the role of ZAG in PPARγ‐regulated ketogenesis in neurons. Neurons were divided into 6 groups: Vector‐*AZGP1*+DMSO, Vector‐RNAi+DMSO, LV‐*AZGP1*+DMSO, LV‐*AZGP1*+GW9662, LV‐*AZGP1*‐RNAi+DMSO and LV‐*AZGP1*‐RNAi+pioglitozole. Neurons were treated with DMSO, GW9662 or pioglitozole for 24 hours and then treated with PA (100 μmol/L) and LC (1 mmol/L) for 8 hours. ZAG expression and AcAc levels were measured.

**Figure 3:**
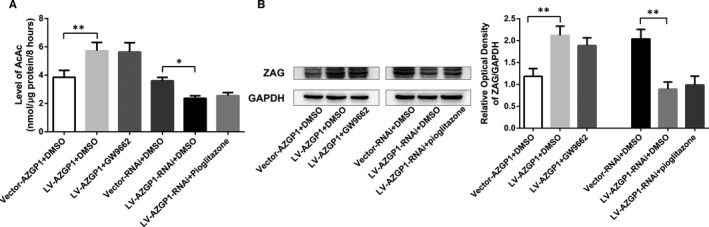
PPARγ regulated ketogenesis by modulating ZAG expression. A, AcAc levels in supernatant of cultured primary neurons were significantly increased in the LV‐*AZGP1*+DMSO group (n = 4) compared to the Vector‐*AZGP1*+DMSO group. This increase in AcAc levels was not abolished by GW9662 (n = 4). AcAc levels were significantly decreased in the LV‐*AZGP1*‐RNAi+DMSO group (n = 4) compared to the Vector‐RNAi+DMSO group. This decrease in the AcAc level was not rescued by pioglitazone (n = 4). B, No significant difference in ZAG protein levels was observed between the LV‐*AZGP1*+DMSO group and LV‐*AZGP1*+GW9662 group (n = 5), or between the LV‐*AZGP1*‐RNAi+DMSO group and LV‐*AZGP1*‐RNAi+pioglitazone group (n = 5). The optical density was normalized to GAPDH levels (* indicates *P* < 0.05, ** indicates *P* < 0.01)

No significant difference in ZAG protein levels was observed between the LV‐*AZGP1*+DMSO group and LV‐*AZGP1*+GW9662 group (2.118 ± 0.213 vs 1.886 ± 0.178, *P* = 0.234, n = 5), or between the LV‐*AZGP1*‐RNAi+DMSO group and LV‐*AZGP1*‐RNAi+pioglitazone group (0.893 ± 0.162 vs 0.987 ± 0.202, *P* = 1.00, n = 5). (Figure 3).

AcAc levels were significantly increased after LV‐*AZGP1* treatment (5.725 ± 0.589 nmol/μg/8 hours vs 3.850 ± 0.486 nmol/μg/8 hours, *P* < 0.001, n = 4) compared to the Vector‐*AZGP1*+DMSO group; this increase in the AcAc level was not abolished by GW9662 (5.635 ± 0.656 nmol/μg/8 hours vs 5.725 ± 0.589 nmol/μg/8 hours, *P* = 1.000, n = 4). In contrast, the AcAc level was significantly decreased after LV‐*AZGP1*‐RNAi treatment (2.366 ± 0.177 nmol/μg/8 hours vs 3.603 ± 0.244 nmol/μg/8 hours, *P* = 0.013, n = 4) compared to the Vector‐RNAi+DMSO group. This decrease in the AcAc level was not rescued by pioglitazone (2.547 ± 0.220 nmol/μg/8 hours vs 2.366 ± 0.177 nmol/μg/8 hours, *P* = 1.000, n = 4), suggesting that the effect of PPARγ on neuronal ketogenesis was mediated by modulating ZAG expression (Figure 3).

### 3.4 ZAG was located in the mitochondria and possibly enhanced HADHB activity by binding to HADHB

We screened potential proteins that interacted with ZAG in primary cultures of cortical neurons using IP/MS to further explore the mechanism by which ZAG affected ketogenesis. After excluding proteins that were precipitated by IgG, 114 proteins precipitated by the anti‐ZAG antibody from 3 independent experiments were identified, including cytosolic proteins, nuclear proteins, ribosomal proteins and mitochondrial proteins. These proteins are involved in protein localization and metabolic processes, among other processes (Table S1).

Using IP/MS, HADHB, a mitochondrial protein required for fatty acid oxidation in the mitochondria, was identified and had a high protein Qscore. We further tested the binding between endogenous ZAG and endogenous HADHB using a coimmunoprecipitation (Co‐IP) assay and found that ZAG coimmunoprecipitated with HADHB. The subsequent reciprocal experiment using an anti‐HADHB antibody also validated this result, indicating an interaction between ZAG and HADHB. The localization of ZAG in mitochondria was examined using double‐labelled immunofluorescence staining with the mitochondrial marker MitoTracker Red and Western blots of mitochondrial proteins extracted from neurons. ZAG colocalized with MitoTracker Red. Western blots also revealed the presence of ZAG in mitochondrial protein extracts along with COX4, a positive marker of mitochondria, but not β‐actin. These findings suggested the localization of ZAG in mitochondria.

**Figure 4:**
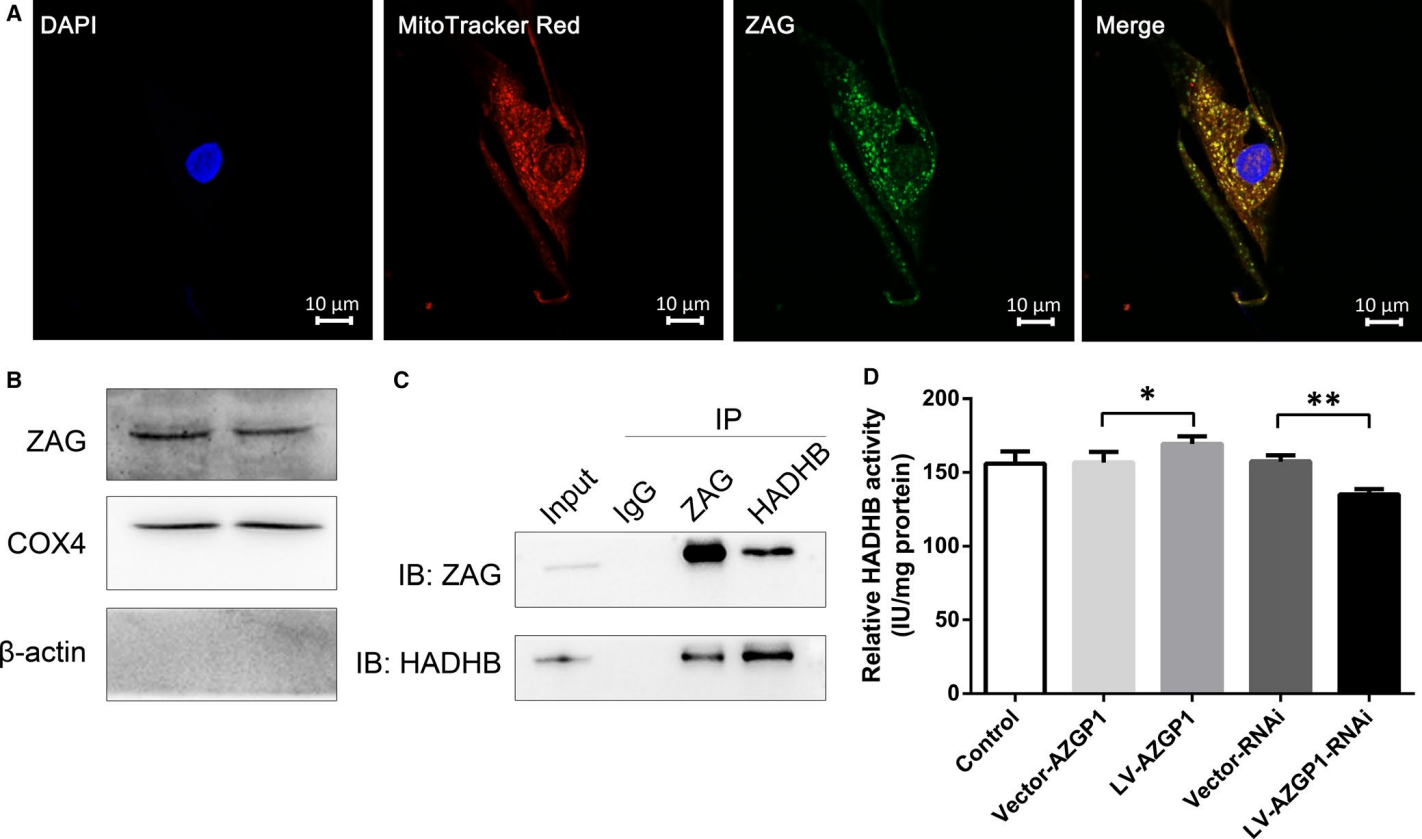
ZAG was located in the mitochondria and increased HADHB activity, possibly by binding to HADHB. A, Cultured primary cortical neurons were stained with the mitochondrial marker MitoTracker (red), DAPI (blue) and anti‐ZAG antibodies (green). Merged images indicate the colocalization of ZAG and mitochondria (yellow). B, Western blots of mitochondrial proteins extracted from cultured cortical neurons. COX4 served as a positive marker of mitochondria, and β‐actin served a negative marker of mitochondria. C, Co‐IP of ZAG with HADHB in cultured cortical neuron lysates. ZAG coimmunoprecipitated with HADHB, and the reciprocal experiment using an anti‐HADHB antibody also validated this result. D, The 3‐ketoacyl‐CoA thiolase activity of HADHB in cultured primary cortical neuron lysates. LV‐*AZGP1*‐RNAi group had significantly decreased 3‐ketoacyl‐CoA thiolase activity of HADHB (n = 5) compared to Vector‐RNAi group, whereas LV‐*AZGP1* group had significantly increased 3‐ketoacyl‐CoA thiolase activity of HADHB compared with Vector‐*AZGP1* group (n = 5). Scale bar indicates 10 μm. (* indicates *P* < 0.05, ** indicates *P* < 0.01)

Considering the role of ZAG in fatty acid metabolism^36^ and limited number of reports on mitochondrial targets of ZAG, we further investigated the effect of ZAG on HADHB activity. LV‐*AZGP1*‐RNAi treatment in neurons significantly decreased the 3‐ketoacyl‐CoA thiolase activity of HADHB (135.005 ± 3.787 IU/mg protein vs 157.360 ± 4.216 IU/mg protein, *P* < 0.001, n = 5) compared to Vector‐RNAi‐transfected neurons, while LV‐*AZGP1* treatment in neurons significantly increased the 3‐ketoacyl‐CoA thiolase activity of HADHB compared with the Vector‐*AZGP1* (169.341 ± 5.076 IU/mg protein vs 156.755 ± 7.122 IU/mg protein, *P* = 0.031, n = 5), suggesting that ZAG increased the 3‐ketoacyl‐CoA thiolase activity of HADHB (Figure 4). Therefore, ZAG may promote ketogenesis in neurons by increasing HADHB activity.

### 3.5 ZAG translocation into the mitochondria was mediated by a HSC70‐dependent mechanism that affected ketogenesis

**Figure 5:**
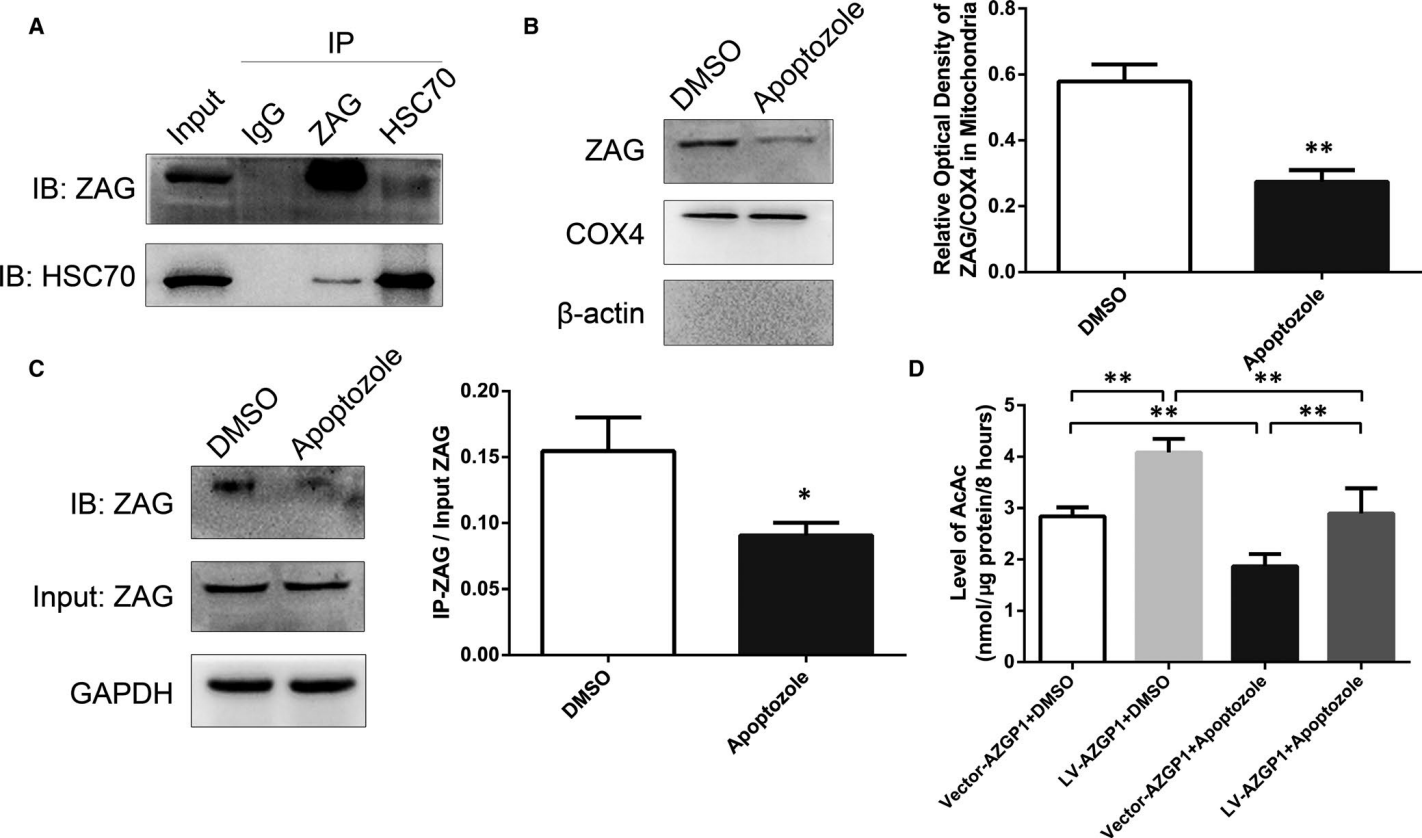
ZAG translocation into the mitochondria was mediated by a HSC70‐dependent mechanism that affected ketogenesis. A, Co‐IP of ZAG with HSC70 in cultured cortical neuron lysates. ZAG coimmunoprecipitated with HSC70, and the reciprocal experiment using the anti‐HSC70 antibody also validated this result. B, Quantification of ZAG level by Western blots of mitochondrial proteins extracted from cultured primary cortical neurons. Compared to the DMSO group, ZAG levels were significantly decreased in the apoptozole‐treated group (n = 5). The optical density was normalized to COX4 levels. C, Quantitative Co‐IP for detecting the interaction between ZAG and HSC70 in cultured cortical neurons treated with the HSC70 inhibitor apoptozole (5 μmol/L) or DMSO. The results showed that the ZAG/GAPDH input was not changed by HSC70 inhibition, but HSC70 inhibition decreased the level of ZAG that was coimmunoprecipitated with HSC70 using the anti‐HSC70 antibody (n = 3). D, AcAc levels in supernatants of cultured primary cortical neurons were significantly decreased in the Vector‐*AZGP1*+apoptozole group than in the Vector‐*AZGP1*+DMSO group. LV‐*AZGP1*+DMSO group had significantly increased AcAc levels compared to the vecto‐*AZGP1*+DMSO group, while this increase was abolished by apoptozole (n = 4) (* indicates *P* < 0.05, ** indicates *P* < 0.01)

HSC70 also interacted with ZAG in IP/MS experiments (Table S2). HSC70 belongs to the heat shock protein family, the structure of which consists of three parts: an ATP‐binding domain, peptide‐binding domain and carboxy‐terminal domain.^37^ HSC70 is abundantly expressed in the mammalian nervous system, particularly in neuronal cell bodies^38^ and is mostly located in the cytoplasm.^39^ As a molecular chaperone, HSC70 possesses a shuttling capacity that drives protein translocation into the mitochondria by binding to target proteins in an ATP‐dependent manner.^16^, ^17^, ^18^, ^19^ The interaction between endogenous ZAG and endogenous HSC70 was tested using a Co‐IP assay. ZAG coimmunoprecipitated with HSC70, and a subsequent reciprocal experiment using an anti‐HSC70 antibody also validated this result (Figure 5A). Neurons were treated with the HSC70 inhibitor apoptozole (5 μmol/L) or DMSO for 24 hours, and then, mitochondrial protein extracts were examined using Western blots to detect the effect of HSC70 on mitochondrial ZAG levels and verify whether the interaction between ZAG and HSC70 was necessary for the mitochondrial translocation of ZAG. Compared to the control, mitochondrial ZAG levels were significantly decreased in the apoptozole‐treated group (0.275 ± 0.035 vs 0.579 ± 0.051, *P* < 0.001, n = 5) (Figure 5B). Quantitative Co‐IP was also used to determine the effects of HSC70 on mitochondrial ZAG levels. The amount of ZAG bound to HSC70 was significantly decreased by the apoptozole treatment compared to DMSO (0.091 ± 0.009 vs 0.155 ± 0.026, *P* = 0.015, n = 3), while apoptozole did not change the total ZAG level compared to DMSO (Figure 5C), indicating that decreased ZAG levels in the mitochondria were a consequence of an altered distribution induced by apoptozole. ZAG binding to HSC70 may be a possible mechanism by which ZAG translocated into the mitochondria.

Additionally, cultured cortical neurons were divided into 4 groups to detect the effect of HSC70‐mediated ZAG translocation on neuronal ketogenesis: Vector‐*AZGP1*+DMSO, LV‐*AZGP1*+DMSO, Vector‐*AZGP1*+apoptozole and LV‐*AZGP1*+apoptozole. Significantly lower neuronal AcAc levels were observed in the Vector‐*AZGP1*+apoptozole group compared to the Vector‐*AZGP1*+DMSO group (1.865 ± 0.242 nmol/μg/8 hours vs 2.837 ± 0.178 nmol/μg/8 hours, *P* = 0.005, n = 4). LV‐*AZGP1* significantly increased AcAc levels compared to the Vector‐*AZGP1*+DMSO group (4.087 ± 0.261 nmol/μg/8 hours vs 2.837 ± 0.178 nmol/μg/8 hours, *P* = 0.001, n = 4), while this increase was abolished by apoptozole (2.898 ± 0.487 nmol/μg/8 hours vs 4.087 ± 0.261 nmol/μg/8 hours, *P* = 0.001, n = 4). Based on these findings, HSC70 regulated the mitochondrial translocation of ZAG and subsequently affected ketogenesis (Figure 5D).

## 4 DISCUSSION

In this study, PTZ‐kindled rats or Mg^2+^‐free ACSF‐treated neurons exhibited impaired ketogenesis characterized by decreased AcAc synthesis and unchanged BHB levels. Seizure‐induced impairments in ketogenesis in both brain tissues from PTZ‐kindled rats and Mg^2+^‐free ACSF‐treated neurons were alleviated by ZAG overexpression, which may be translocated into mitochondria by HSC70 and increase the activity of HADHB in mitochondria, an enzyme known to promote fatty acid β‐oxidation. ZAG expression in neurons may be regulated by PPARγ.

In the present study, brain tissues from PTZ‐kindled rats or cultured neurons treated with Mg^2+^‐free ACSF showed decreased KB level and ketogenesis. KBs are known to suppress epilepsy or seizures through various mechanisms for a long time, including the inhibition of glutamatergic transmission, reduction in neuronal excitability, and induction of GABA synthesis.^5^, ^6^, ^7^, ^8^ However, for the first time, KBs, specifically AcAc, were found decreased in seizure models both in vivo and in vitro, indicating a role of ketogenesis deficiency in the pathophysiology of epilepsy or seizure.

Interestingly, seizure only reduced the synthesis of AcAc, while the BHB level was unchanged. In a study focused on KB metabolism in cultured neurons and astrocytes, [3‐^14^C]AcAc was used to calculate the rate of AcAc transformation to BHB, and the amount of BHB produced from [3‐^14^C]AcAc was <5% of the decrease in AcAc concentration in neurons and 10%‐22% in astrocytes, indicating a very low degree of transformation of AcAc into BHB in cultured neurons.^40^ In mouse brain, the rate of AcAc oxidation was two‐fold higher than BHB, and the rate of lipogenesis of BHB accounted for less than 10% of AcAc.^41^ These data indicated a preference for AcAc utilization in brain and neuron, and may partially explain the difference effect of seizure on AcAc synthesis and BHB synthesis. Similarly, blood BHB levels are unchanged in epileptic mice fed on standard diet or ketogenic diet (KD), but the authors did not measure KB levels in brain tissues.^42^ Consequently, our findings indicated that AcAc may be the major KB component affected by epilepsy or seizure, possibly due to the preference of neurons for AcAc.

Consistent with our previous studies,^10^, ^43^ ZAG was located in neurons both in vitro and in vivo, and seizures induced by PTZ kindling in vivo or Mg^2+^‐free ACSF treatment in vitro significantly decreased neuronal ZAG expression. As a lipid‐mobilizing factor, ZAG promotes lipolysis, fatty acid β‐oxidation and ketogenesis in hepatocytes and adipocytes.^13^, ^44^, ^45^ Similarly, in neurons, we found ZAG promoted ketogenesis, and ZAG overexpression alleviated epilepsy/seizure‐induced impairments in neuronal ketogenesis, while knockdown of ZAG suppressed ketogenesis in cultured neurons, similar to the Mg^2+^‐free ACSF treatment, supporting the role of ZAG in seizure‐induced impairment in ketogenesis. Therefore, it is possible that seizures impair neuronal ketogenesis by reducing ZAG expression, and ZAG likely exerts its anti‐epileptic/seizure effect by enhancing ketogenesis.

In this study, we further explored the potential mechanism by which ZAG regulates neuronal ketogenesis using IP/MS, and found ZAG bound to HADHB. HADHB is a mitochondrial protein with long‐chain 3‐ketocayl‐CoA thiolase activity that catalyses the fourth step of long‐chain fatty acid β‐oxidation ^46^ and promotes acetyl coenzyme A production,^1^ subsequently promoting ketogenesis. The binding between ZAG and HADHB in the mitochondria was verified by double‐labelled immunofluorescence staining, Western blots and Co‐IP. Furthermore, ZAG overexpression increased HADHB activity, while ZAG knockdown decreased HADHB activity. This report may be the first to describe the existence and role of ZAG in the mitochondria and provide a possible mechanism that ZAG regulates neuronal ketogenesis by modulating HADHB. However, the specific mechanism by which ZAG regulates HADHB activity requires further research. The effect of ZAG‐regulated HADHB activity on neuronal ketogenesis also needs further investigation.

As a plasma protein, the mechanism by which ZAG enters the mitochondria may also play important role in regulation of neuronal ketogenesis, however, this mechanism has never been elucidated. In IP/MS, ZAG was found to bind a chaperone protein, HSC70. HSC70 facilitates protein translocation into mitochondria.^16^, ^17^, ^18^, ^19^ We found that inhibition of HSC70 by apoptozole suppressed the interaction between ZAG and HSC70, and decreased ZAG levels in mitochondria, revealing an association between the binding of ZAG to HSC70 and the translocation of ZAG into mitochondria. Additionally, the inhibition of HSC70 activity also suppressed the effect of ZAG overexpression on promoting ketogenesis, indicating that ZAG‐regulated neuronal ketogenesis depends on ZAG translocation into mitochondria. Interestingly, BHB has been reported to inhibit HDACs,^47^ which suppress HSC70 activity by inhibiting deacetylation.^48^ Therefore, a potential negative feedback loop may exist between KBs and ZAG translocation, which may sustain KB levels in neurons. However, direct evidence for this feedback loop is not available, and in the present study, we failed to link BHB levels to neuronal ZAG expression. Researchers have not clearly determined whether AcAc exerts a similar effect on HSC70 deacetylation. The specific role and mechanism of ZAG in neuronal fatty acid metabolism and ketogenesis require further study.

The mechanism by which epilepsy/seizures decrease ZAG expression has never been elucidated. We found in neurons that PPARγ could bind to the promoter region of *AZGP1*, and pioglitazone‐induced PPARγ activation increased ZAG expression in neurons, while PPARγ inhibition by GW9662 suppressed ZAG expression. These findings are consistent with previous studies of ZAG levels in the circulation and adipocytes of patients.^20^, ^21^ However, a luciferase reporter assay is needed to further verify the specific effect of PPARγ binding on *AZGP1* gene transcription. PPARγ levels are decreased in brain tissues from epileptic mice,^22^ and PPARγ exerts an anti‐epileptogenic effect.^49^, ^50^ It is possible that seizures suppress ZAG expression by reducing PPARγ levels, but the effect of PPARγ on the seizure‐induced decrease in ZAG levels remains to be verified. Additionally, in the present study, PPARγ activation‐promoted neuronal ketogenesis was abolished by ZAG knockdown. Similarly, the inhibitory effect of GW9662, an antagonist of PPARγ, on ketogenesis was also abolished by ZAG overexpression. It is possible that PPARγ promotes neuronal ketogenesis by increasing ZAG expression. As PPARγ is also reported to promote ketogenesis by increasing HMGCS2, BDH1 and PDK4 levels,^26^ ZAG might regulate neuronal ketogenesis directly or indirectly by modulating the levels of these 3 enzymes, but evidence is unavailable. Notably, ZAG may also regulate ketogenesis by inducing the expression of PPARγ ^51^; therefore, a feedback loop may exist between ZAG and PPARγ. However, the effect of ZAG on PPARγ expression is controversial, because ZAG has also been reported to inhibit PPARγ expression.^52^

In clinic, ketogenic diet (KD) is regarded as an effective treatment for refractory epilepsy.^53^, ^54^ The mechanism underlying the anti‐epileptic effect of KD has not completely elucidated, but KBs are mostly considered mediators of the anti‐epileptic effect of KD therapy.^1^, ^55^ However, KD induces hyperketonemia and a low‐grade systemic chronic metabolic acidosis,^56^ resulting in short‐ and long‐term systemic adverse effects and an increased risk of early mortality, which limit the use of KD to treat epilepsy. Thus, strategies that merely modulate neuronal ketogenesis may be an alternative to KD for treating epilepsy without increasing KB levels in the circulation. Additionally, KD was reported not beneficial to patients with epileptiform discharges in the temporal region,^57^ and our study was also performed in rat cerebral cortex and cultured cortical neurons rather than hippocampus, an important structure in the pathophysiology and pathogenesis of temporal lobe epilepsy. Therefore, it is possible that effect of KBs on seizure/epilepsy may depend on epileptogenic zone.

The present study is the first to show that KBs were decreased in brain tissues and neurons after seizure due to impairment of neuronal ketogenesis induced by seizure. One mechanism that seizures decrease neuronal ketogenesis was decreasing ZAG in neurons. The regulatory effect of ZAG on ketogenesis was mediated by the HSC70‐dependent transfer of ZAG into the mitochondria and may rely on its effect on enhancing HADHB activity. These findings proposed a mechanism of fatty acid metabolism and ketogenesis in neurons and may improve understanding of neuronal metabolism.

## CONFLICT OF INTEREST

The authors confirm that there are no conflicts of interest.

## AUTHORS’ CONTRIBUTIONS

LC, YC, CT and XL conceived the study. CT, XL and WP designed the methodology. LC, CT and XL performed the formal analysis. CT, XL, WP, HW, WZ, JJ, XW and LM investigated the study. CT and XL wrote the original draft of the manuscript. LC and YC wrote, reviewed and edited the manuscript. CT and XL visualized the study. LC and YC supervised the study. LC, YC and XL administered the project. LC involved in funding acquisition. All authors read and approved the final manuscript.

## Supporting information

Supplementary Material

## Data Availability

All data generated or analysed during this study are included in this published article.
