# Supplementary material for: Seizure‐induced impairment in neuronal ketogenesis: Role of zinc‐α2‐glycoprotein in mitochondria
Source: J Cell Mol Med. 2020 Apr 27;24(12):6833–45. doi: 10.1111/jcmm.15337 (PMC7299723; doi:10.1111/jcmm.15337)
Supplement: Supplementary file 1 — Supplementary Material [file JCMM-24-6833-s001.pdf]

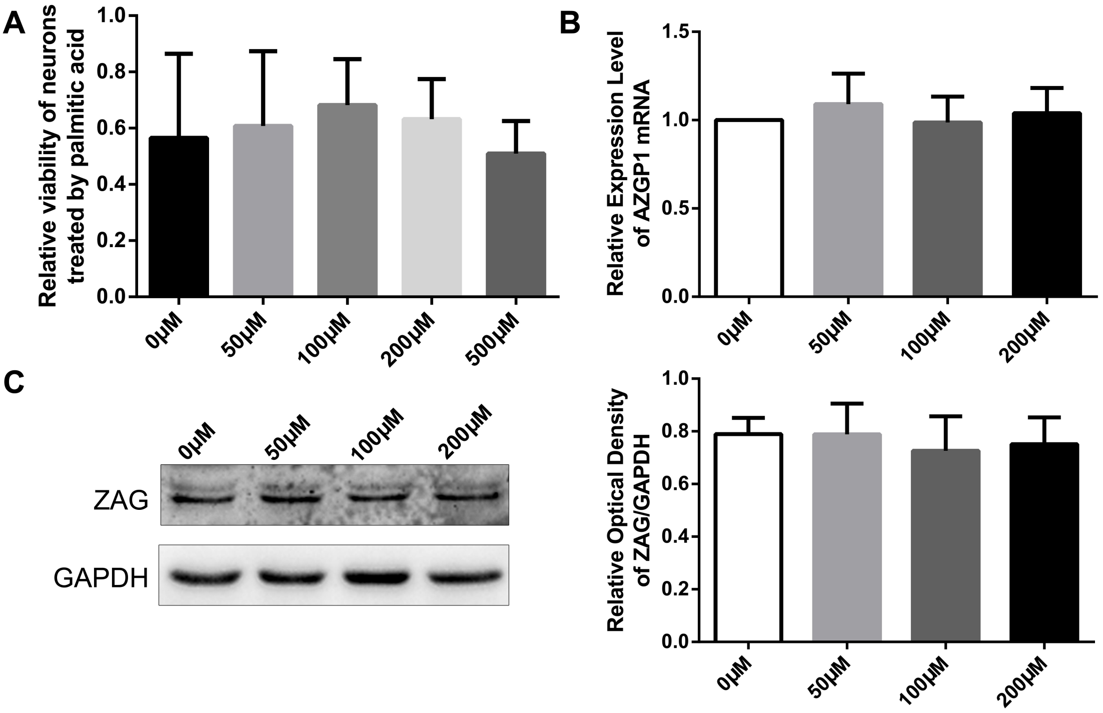

Figure S1. The PA treatment did not alter neuronal viability or ZAG expression in cultured neurons. Related to Figure 1-3 and 5.

(A) The MTS method was used to measure the viability of cultured neurons treated with 0, 50, 100, 200 or 500 μM PA for 8 hours. A concentration of 100 μM was chosen for subsequent experiments.

(B) Quantification of the expression of the *AZGP1* mRNA in cultured cortical neurons treated with different concentrations of PA and LC (1 mM) for 8 hours using qRT-PCR. Treatment with 0, 50, 100 and 200 μM PA did not change the expression of the AZGP1 mRNA (n=3).

(C) Quantification of ZAG expression using western blots of cultured cortical neurons treated with different concentrations of PA and LC (1 mM) for 8 hours. At concentrations of 0, 50, 100 and 200 μM, PA did not alter the levels of the ZAG protein (n=5). The optical density was normalized by GAPDH levels. * $p<0.05$, ** $p<0.01$.

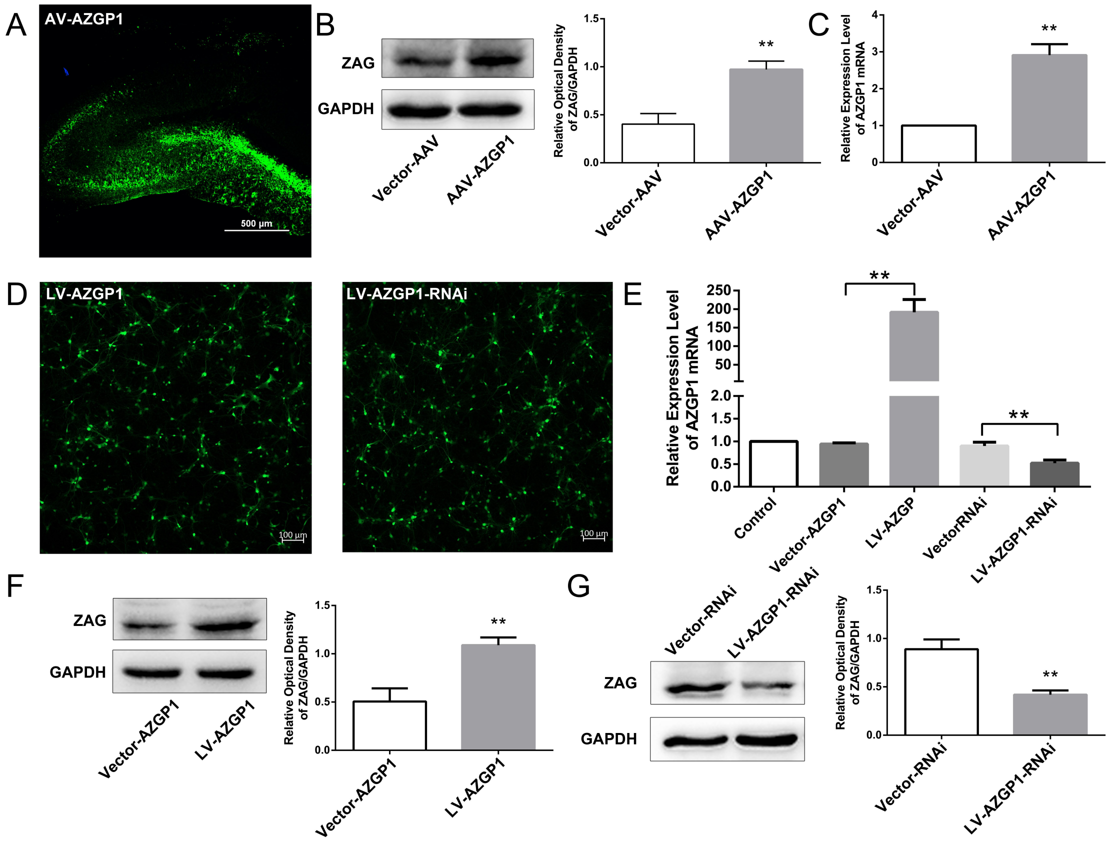

Figure S2. Changes in the expression of GFP after the injection of AAV in rats and after the transfection of LV-*AZGP1*, LV-*AZGP1*-RNAi, Vector-*AZGP1*, and Vector-RNAi in neurons. Related to Figure 1.

(A) Fluorescence images showing GFP expression in the hippocampus of rats at 3 weeks after the AAV injection.

(B) Quantification of ZAG expression using western blots of proteins extracted from rat brain tissues after the AAV injection. Levels of the ZAG protein in the cortex revealed the successful overexpression of (AAV)-AZGP1 compared to rats that received the vector injection (n=4). The optical density was normalized to GAPDH levels.

(C) Quantification of the expression of the *AZGP1* mRNA in brain tissues from rats injected with AAV using qRT-PCR. The expression of the *AZGP1* mRNA in the cortex revealed the successful overexpression following the AAV-*AZGP1* injection compared to rats that received the vector injection (n=3).

(D) Fluorescence images showing GFP expression in the cultured cortical neurons transfected with LV.

(E) Quantification of the expression of the *AZGP1* mRNA in cultured cortical neurons transfected with LV using qRT-PCR. LV-*AZGP1* significantly increased the expression of the *AZGP1* mRNA (n=3), whereas LV-*AZGP1*-RNAi decreased the expression of the *AZGP1* mRNA (n=3).

(F) Quantification of ZAG expression using western blots of proteins extracted from cultured primary cortical neurons transfected with LV. LV-*AZGP1* significantly increased levels of the ZAG protein (n=4). The optical density was normalized to GAPDH levels.

(G) Quantification of ZAG expression using western blots of proteins extracted from cultured primary cortical neurons transfected with LV. LV-*AZGP1*-RNAi decreased levels of the ZAG protein (n=4). The optical density was normalized to GAPDH levels. * $p<0.05$, ** $p<0.01$.

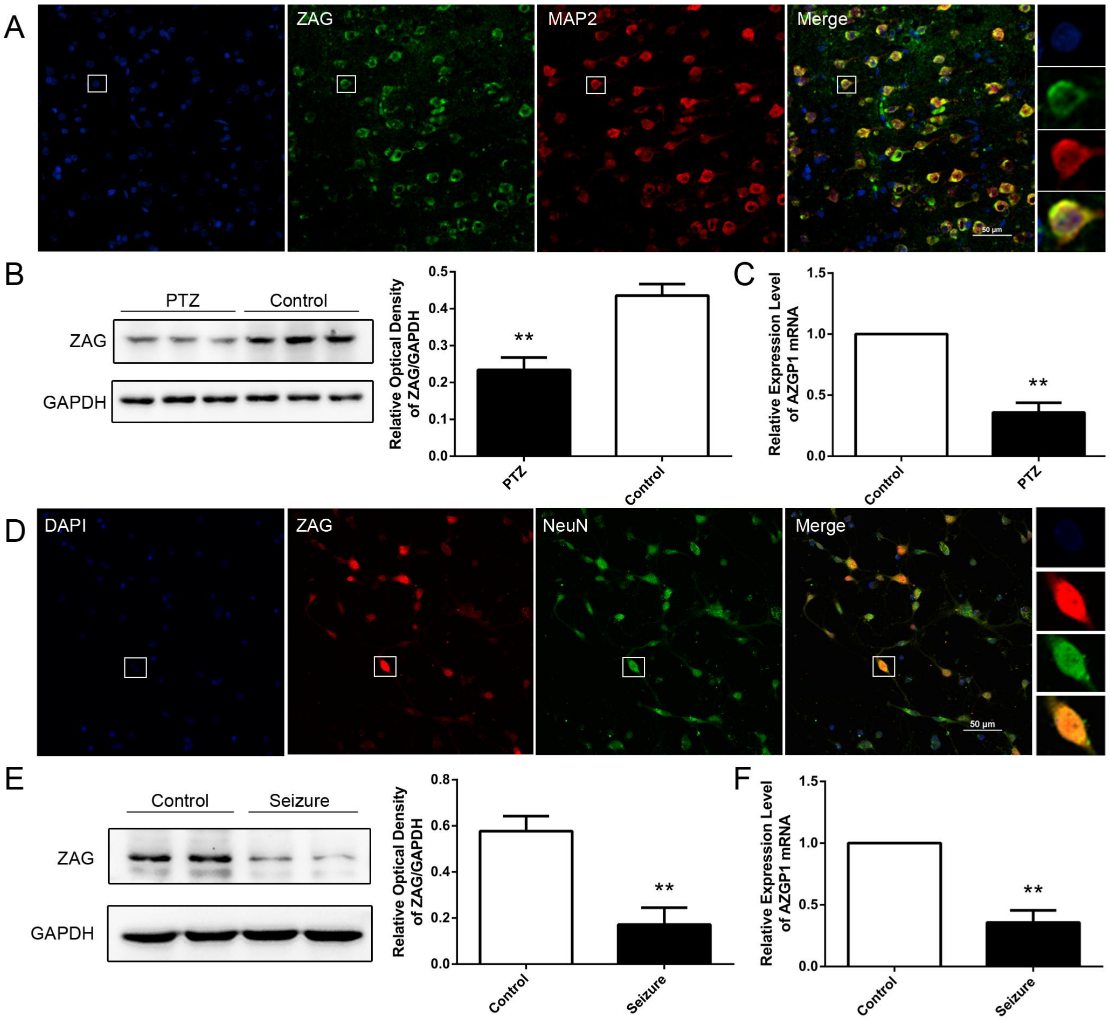

Figure S3. Neuronal ZAG expression was decreased by seizures both in vivo and in vitro. Related to Figure 1.

(A) Localization of ZAG in neurons from rat brain sections. Rat brain sections were stained with 4'-6-diamidino-2-phenylindole (in blue), the neuronal marker MAP2 (red), and antibodies against ZAG (green). Confocal imaging was performed using an A1+R laser confocal microscope. Merged images indicate the colocalization of ZAG and neurons (yellow).

(B) Quantification of ZAG expression using western blots of proteins extracted from brain tissues from PTZ-kindled rats. Levels of the ZAG protein in the PTZ-kindled rat cortex were decreased compared to the control (n=4). The optical density was normalized to GAPDH levels.

(C) Quantification of *AZGP1* mRNA expression in brain tissues from PTZ-kindled rats using qRT-PCR. The expression of the *AZGP1* mRNA was decreased in the PTZ-kindled rat cortex were compared to the control (n=3).

(D) Localization of ZAG in cultured primary cortical neurons. Neurons were stained with 4'-6-diamidino-2-phenylindole (blue), the neuronal marker NeuN (green), and antibodies against ZAG (red). Confocal imaging was performed using an A1+R laser confocal microscope. Merged images indicate the colocalization of ZAG and neurons (yellow).

(E) Quantification of ZAG expression using western blots of proteins extracted from cultured primary cortical neurons that were treated with Mg2+-free ACSF for 3 hours. Levels of the ZAG protein were

decreased in the seizure group compared to the control (n=4). The optical density was normalized to GAPDH levels.

(F) Quantification of the expression of the *AZGP1* mRNA in cultured primary cortical neurons treated with Mg2+-free ACSF for 3 hours using qRT-PCR. The expression of the *AZGP1* mRNA was decreased in the seizure group compared to the control (n=3). * $p<0.05$, ** $p<0.01$.

Tabel S1. Gene ontology analysis of zinc-α2-glycoprotein-associated proteins. Related to Figure 4.

Biological Process	FDR	Genes input	Genes in Genome
actin polymerization or depolymerization	4.62E-02	4	65
actin filament organization	1.58E-03	7	129
cytoplasmic translation	3.62E-02	5	110
cytoskeleton organization	4.93E-04	10	228
microtubule-based process	2.09E-03	8	193
actin cytoskeleton organization	7.38E-03	7	180
formation of translation initiation ternary complex	1.48E-03	10	294
translational termination	1.27E-03	10	294
translational elongation	1.11E-03	10	294
translation	2.38E-03	10	334
organelle organization	9.38E-04	18	835
intracellular protein transport	7.58E-03	13	663
cellular localization	3.42E-03	16	892
vesicle-mediated transport	4.39E-02	11	616
cellular protein localization	1.09E-02	14	798
cellular macromolecule localization	1.07E-02	14	801
cellular component organization	5.76E-04	26	1712
localization	4.47E-03	26	2041
cellular process	4.61E-04	57	6051
**Cellular Component**	**FDR**	**Genes input**	**Genes in Genome**
cytoplasmic dynein complex	4.90E-02	2	11
cytoskeletal part	6.55E-03	4	51
actin cytoskeleton	7.30E-06	11	215
cytosolic large ribosomal subunit	6.62E-04	9	247
cytoskeleton	6.58E-08	19	550
microtubule	4.13E-02	5	156
cytosolic ribosome	1.75E-03	10	366
cytosolic part	1.02E-03	11	414
intracellular non-membrane-bounded organelle	7.33E-05	21	1139
non-membrane-bounded organelle	5.87E-05	21	1139
cytosol	4.36E-03	16	1018
protein-containing complex	6.14E-03	20	1554
cytoplasm	2.05E-03	39	3875
intracellular part	5.73E-03	39	4079
organelle	2.05E-02	35	3765
cell part	4.03E-03	51	5919
cell	4.93E-03	51	5943

intracellular	3.50E-02	40	4690
Molecular Function	FDR	Genes input	Genes in Genome
actin filament binding	1.78E-05	7	65
actin binding	2.49E-04	8	150
cytoskeletal protein binding	4.68E-08	16	349
structural constituent of ribosome	1.93E-03	10	365
structural molecule activity	1.53E-05	18	719
RNA binding	1.23E-03	14	677
protein binding	1.13E-03	32	2729
binding	2.49E-04	53	5516
PANTHER Pathways	FDR	Genes input	Genes in Genome
ATP synthesis	4.87E-02	2	11
Cytoskeletal regulation by Rho GTPase	3.22E-05	7	80
Parkinson disease	2.51E-05	8	105
Cadherin signaling pathway	7.22E-04	7	140
Huntington disease	3.09E-03	7	186
Inflammation mediated by chemokine and cytokine signaling pathway	1.68E-02	7	257

Table S2. Zinc-α2-glycoprotein-associated peptides. Related to Figure 4 and 5.

Gene	Protein	Accession Number	Number of peptides	Coverage (%)	Qscore
Tjp1	Tight junction protein ZO-1	A0A0G2K2P5	1	0.62	2.37
Tollip	Toll-interacting protein	A2RUW1	2	9.85	6.08
Brsk1	Serine/threonine-protein kinase BRSK1	B2DD29	2	3.47	5.61
Macf1	Microtubule-actin cross-linking factor 1	D3ZHV2	4	1.01	10.19
Kif21b	Kinesin-like protein KIF21B	F1M5N7	1	1.04	2.84
Hnrnpc	Heterogeneous nuclear ribonucleoprotein C	G3V9R8	1	3.69	3.04
Gja1	Gap junction alpha-1 protein	P08050	2	6.02	6.08
Npm1	Nucleophosmin	P13084	3	18.49	8.88
Ap2a2	AP-2 complex subunit alpha-2	P18484	5	6.29	14.42
Csnk2a1	Casein kinase II subunit alpha	P19139	7	23.79	21.10
Gnaz	Guanine nucleotide-binding protein G(z) subunit alpha	P19627	1	4.79	2.84
Rpl6	60S ribosomal protein L6	P21533	3	15.1	7.95
Ppib	Peptidyl-prolyl cis-trans isomerase B	P24368	1	6.02	2.42
Dync1h1	Cytoplasmic dynein 1 heavy chain 1	P38650	8	2.3	22.77
Rpsa	40S ribosomal protein SA	P38983	2	10.85	5.88
Rps3a	40S ribosomal protein S3a	P49242	3	15.91	8.63
Gpc2	Glypican-2	P51653	1	2.94	3.04
Gnb1	Guanine nucleotide-binding protein G(I)/G(S)/G(T) subunit beta-1	P54311	1	8.82	2.79
Rpl15	60S ribosomal protein L15	P61314	1	5.88	3.04
Rpl27	60S ribosomal protein L27	P61354	2	22.06	5.32
Ppp1ca	Serine/threonine-protein phosphatase PP1-alpha catalytic subunit	q111111	1	13.64	4.81
Ran	GTP-binding nuclear protein Ran	P62828	3	19.91	14.43
Rpl23	60S ribosomal protein L23	P62832	1	10.71	5.14
Rpl8	60S ribosomal protein L8	P62919	1	4.28	2.32
Dnaja1	DnaJ homolog subfamily A member 1	P63036	1	4.79	2.84
Actc1	Actin, alpha cardiac muscle 1	P68035	5	48.01	12.39
Tmod2	Tropomodulin-2	P70566	4	19.09	11.72
Actr1a	Alpha-centractin	P85515	1	5.85	3.04
Arpc2	Actin-related protein 2/3 complex subunit 2	P85970	6	21	30.67
Slc27a1	Long-chain fatty acid transport protein 1	P97849	1	2.01	2.29
Eif3a	Eukaryotic translation initiation factor 3 subunit A	Q1JU68	3	3.25	8.16
Ubr4	E3 ubiquitin-protein ligase UBR4	Q2TL32	1	0.54	3.04
Atad3	ATPase family AAA domain-containing protein 3	Q3KRE0	2	6.77	5.34
Hnrnpdl	Heterogeneous nuclear ribonucleoprotein D-like	Q3SWU3	1	2.48	2.79
Capza2	F-actin-capping protein subunit alpha-2	Q3T1K5	3	27.97	9.12
Wdr1	WD repeat-containing protein 1	Q5RKI0	1	3.47	2.90
Rnmt	mRNA cap guanine-N7 methyltransferase	Q5U2U7	1	4.99	2.79
Hadhb	Trifunctional enzyme subunit beta, mitochondrial	Q60587	4	10.32	11.35

Fyn	Tyrosine-protein kinase Fyn	Q62844	2	6.7	6.08
Dync1i2	Cytoplasmic dynein 1 intermediate chain 2	Q62871	1	3.45	2.79
Cask	Peripheral plasma membrane protein CASK	Q62915	1	1.21	2.11
Map6	Microtubule-associated protein 6	Q63560	1	1.26	3.04
Cttn	Src substrate cortactin	Q66HL2	1	2.55	3.04
Cct5	T-complex protein 1 subunit epsilon	Q68FQ0	1	1.85	3.04
Pa2g4	Proliferation-associated protein 2G4	Q6AYD3	1	4.57	3.04
Eif3d	Eukaryotic translation initiation factor 3 subunit D	Q6AYK8	1	2.01	2.42
Ehd3	EH domain-containing protein 3	Q8R491	1	3.18	2.79
Palm	Paralemmin-1	Q920Q0	2	10.18	4.93
Zbtb18	Zinc finger and BTB domain-containing protein 18	Q9JKY3	1	4.6	2.84
Htra1	Serine protease HTRA1	Q9QZK5	1	2.5	2.34
Flot1	Flotillin-1	Q9Z1E1	1	2.34	2.84
Vdac3	Voltage-dependent anion-selective channel protein 3	A0A0G2JSR0	1	7.07	3.04
Nfia	Nuclear factor 1	A0A0G2JUB7	1	3.73	2.53
Ywhaz	14-3-3 protein zeta/delta	A0A0G2JV65	1	8.2	2.72
LOC103692829	60S ribosomal protein L9 pseudogene	A0A0G2JXD0	2	12.77	5.41
Sptan1	Spectrin alpha chain, non-erythrocytic 1	A0A0G2JZ69	2	24.95	5.74
LOC100911769	Band 4.1-like protein 1	A0A0G2K0B2	1	4.58	2.79
Ank3	Ankyrin-3	A0A0G2K1R9	1	0.9	2.42
Dock7	Dedicator of cytokinesis 7	A0A0G2K3H2	4	2.02	10.59
Rpl11	60S ribosomal protein L11	A0A0G2K3Y8	1	7.45	2.84
Mfge8	Lactadherin	A0A0G2K506	3	9.21	8.22
Tpm1	Tropomyosin alpha-1 chain	A0A0G2K7F7	3	13.86	7.94
Tars	Threonine--tRNA ligase, cytoplasmic	A0A0G2K9V6	1	1.66	2.15
Fscn1	Fascin	A0A0G2KAM4	1	3.25	3.04
Gsk3b	Glycogen synthase kinase-3 beta	A0A0G2KB98	1	5.58	3.04
Rab6a	RCG39700, isoform CRA_d	A0A0H2UHP9	1	5.77	2.84
Nap1l4	Nucleosome assembly protein 1-like 4	A0A0H2UHZ2	2	10.08	4.72
Map2	Microtubule-associated protein	A0A0U1RRX4	3	2.68	7.85
Upf1	UPF1, RNA helicase and ATPase	A0A0U1RS25	1	0.98	2.79
Klc1	Kinesin light chain 1	A0A140TAB3	1	2.36	3.04
Rpl12	60S ribosomal protein L12	A0A140TAC5	2	19.28	5.65
Arpc3	Actin-related protein 2/3 complex subunit 3	B2GV73	1	6.18	3.04
Arpc4	Actin-related protein 2/3 complex subunit 4	B2RZ72	4	30.95	11.97
Csnk2a2	Casein kinase 2 alpha 2	B4F7A9	1	4.29	2.72
Ap3d1	Adaptor-related protein complex 3, delta 1 subunit	B5DFK6	3	3.65	8.88

Agk	Acylglycerol kinase	D3Z9L0	1	5.1	2.72
Epha5	Ephrin type-A receptor 5	D3ZBZ7	1	1.46	2.84
Plxna4	Plexin A4	D3ZES7	1	0.26	2.72
LOC100 909664	Centrosomal protein of 170 kDa-like	D3ZET9	1	1.23	3.04
Lactb	Lactamase, beta	D3ZFJ6	3	8.18	8.40
LOC100 360491	60S ribosomal protein L13	D3ZRM9	1	6.16	3.04
Actbl2	Actin, beta-like 2	D3ZRN3	2	33.78	4.54
Usp5	Ubiquitinyl hydrolase 1	D3ZVQ0	1	1.75	2.79
Mvb12b	Multivesicular body subunit 12B	D4A732	1	7.89	3.04
Hist2h2a c	Histone H2A	D4ACV3	1	15.8	3.04
Ipo7	Importin 7	D4AE96	2	2.7	5.68
Mapk8ip 3	Mitogen-activated protein kinase 8-interacting protein 3	E9PSK7	1	1.89	3.04
Myh11	Myosin-11	E9PTU4	2	6.24	6.08
R	Similar to ribosomal protein S10	F1LT36	1	5.45	3.04
Svil	Supervillin	F1M155	1	0.37	2.79
Map4k4	Mitogen-activated protein kinase kinase kinase kinase 4	F1M754	3	2.97	8.68
Kif2a	Kinesin-like protein	F1M8L1	3	5.26	8.30
Elavl2	ELAV-like protein	G3V6U4	1	3.09	3.04
Glg1	Golgi apparatus protein 1	G3V8G5	2	1.71	5.74
Syncrip	Heterogeneous nuclear ribonucleoprotein Q	M0R735	3	6.58	8.68
Csnk2b	Casein kinase II subunit beta (Fragment)	N0E631	1	15.91	3.04
U2af1	RCG60540, isoform CRA_a	Q3KR55	1	7.53	3.04
Hdlbp	High density lipoprotein binding protein (Vigilin)	Q3KRF2	1	0.71	2.03
Ap2b1	AP complex subunit beta	Q3ZB97	6	7.89	14.96
Asna1	Arsenical pump-driving ATPase (Fragment)	Q4G022	1	3.04	2.90
Tubb6	Tubulin beta chain	Q4QQV0	1	26.62	2.79
Rbm39	RNA binding motif protein 39	Q5BJP4	1	2.1	2.56
Pacsin3	Protein kinase C and casein kinase substrate in neurons 3	Q5I2Z0	1	2.83	2.22
Lars	Leucyl-tRNA synthetase	Q5PPJ6	1	0.59	2.79
Krt15	Keratin, type I cytoskeletal 15	Q6IFV3	1	2.68	2.42
Dsp	Desmoplakin	F1LMV6	1	0.45	2.42
Atp5f1a	ATP synthase subunit alpha	F1LP05	3	6.87	8.88
Hspa8	Heat shock cognate 71 kDa protein	D4A4S3	9	17.98	25.90
Atp5f1b	ATP synthase subunit beta, mitochondrial	P10719	4	11.34	11.16
Gldc	Glycine cleavage system P protein	A0A0G2JUZ5	1	2.01	3.04
Igh-1a	Ig gamma-2B chain C region	P20761	1	4.8	3.04
Cbwd1	COBW domain-containing protein 1	Q99MB4	1	3.55	3.04

Table S3. Primer sequences used in this study.

AZGP1	forward	5′-TTCAAGCCACCGCATTTCTC-3′
	reverse	5′-TCCTTCTCCCAGTCCTCCATTC-3′
GAPDH	forward	5′-ACGGTCAGGTCATCACTATCG-3′
	reverse	5′-GGCATAGAGGTCTTTACGGATG-3′
Promoter region of *AZGP1*	forward (12q11, NC_005111.4 (19195247..19195269))	5’-GAGTGGTGGCAAGAGTCTGAAGC-3’
	reverse (12q11,NC_005111.4 (19203988..19204008))	5’-TGGCGGTGTCTAGGCTCATCG-3’
